# Willingness to pay for packaging cancer screening of Chinese rural residents

**DOI:** 10.1002/cam4.5162

**Published:** 2022-08-24

**Authors:** Qianqian Zhang, Deyu Ren, Xuan Chang, Chen Sun, Ruyue Liu, Jialin Wang, Nan Zhang

**Affiliations:** ^1^ School of Public Health, Cheeloo College of Medicine Shandong University Jinan China; ^2^ Center for Cancer Control and Policy Research (CCPR) Shandong University Jinan China; ^3^ NHC Key Laboratory of Health Economics and Policy Research (Shandong University) Jinan China; ^4^ Department of Publicity Shandong Provincial Hospital Affiliated to Shandong First Medical University Jinan China; ^5^ School of Public Health Weifang Medical University Weifang China; ^6^ Shandong Cancer Hospital and Institute Shandong First Medical University and Shandong Academy of Medical Sciences Jinan China

**Keywords:** contingent valuation method, packaging cancer screening, rural residents, Tobit model, willingness to pay

## Abstract

**Background:**

This study examined the acceptance and willingness to pay (WTP) of rural residents toward packaging cancer screening (PCS) to provide a reference basis for promoting the screening sustainable development.

**Methods:**

A face‐to‐face questionnaire survey was conducted among rural residents aged 40–69. The combination of double‐bounded dichotomous choices and open‐ended questions in the Contingent Valuation Method was used to guide participants' WTP. Logistic regression was used to explore the influencing factors of participants' screening acceptance, and Tobit model was used to analyze the associated factors of WTP.

**Results:**

Of the 959 respondents, 89.36% were willing to accept PCS, but 10.64% stated unwillingness for the dominant reason that they did not attend clinics until symptom onset. Willingness to accept screening was significantly associated with region (Dongchangfu, OR = 0.251, 95%CI: 0.113~0.557; Linqu, OR = 0.150, 95%CI: 0.069~0.325), age with 60–69 (OR = 0.321, 95%CI: 0.126~0.816), annual income with 10,000–30,000 (OR = 1.632, 95%CI: 1.003~2.656) and having cancer‐screening experience (OR = 0.581, 95%CI: 0.371~0.909). And 57.66% of participants were willing to pay part of the screening cost among those willing to accept PCS. The residents' average WTP was ¥622, accounting for 20.73% of the total cost (¥3000). Willingness to pay for PCS was positively correlated with male gender, self‐employed occupation, residence in Feicheng (than Linqu), higher income, and having cancer‐screening experience.

**Conclusions:**

Most rural residents were willing to accept free PCS, more than half of them were willing to pay part of the ¥3000 total cost, but their WTP‐values were low. It is necessary to carry out PCS publicity activities to improve public awareness and participation in precancerous screening. Additionally, for expanding the coverage and sustainability of screening, the appropriate proportion of rural residents to pay for screening costs should be controlled at about 20%, and governments, insurance and other sources are encouraged to actively participate to cover the remaining costs.

## Graphical abstract

This study is the first to examine the acceptance and willingness to pay (WTP) of rural residents toward packaging cancer screening (PCS). We found that most rural residents were willing to accept free PCS, more than half of them were willing to pay part of the ¥3000 total cost, but their WTP values were low. The appropriate proportion of rural residents to pay for screening costs should be controlled at about 20%, and governments, insurance and other sources are encouraged to actively participate to cover the remaining costs.

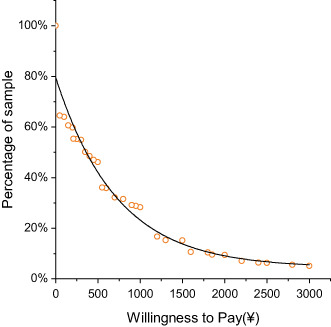

## 1 INTRODUCTION

Cancer leads to the first or second cause of death in most countries. About 28.4 million cancer cases are estimated to occur by 2040, with an increase of 47% compared with 19.3 million in 2020, which has imposed a heavy burden on human health.^1^ The morbidity and mortality of lung cancer,^2^ gastric cancer,^3^ esophageal cancer,^4^ liver cancer,^5^ colorectal cancer,^6^ and breast cancer^7^ have remained at a high level in China, which Disability Adjusted of Life Years (DALYs) had reached more than 65% of all cancer DALYs by 2020.^8^ Studies have demonstrated that patients' prognosis is closely related to the time of initial diagnosis.^9^, ^10^ Unfortunately, symptoms tend to occur only in an advanced stage of the disease, when the prognosis is poor and treatment is expensive. Screening has been proven to be one of the most cost‐effective health investments to improve the detection rate and the survival rate of cancer via moving the cancer diagnosis timing forward.^11^, ^12^, ^13^ Multiple‐site cancer screening (i.e. packaging cancer screening, PCS) can maximize efficiency, explicitly taking into account a limited budget and scarce resources, constraints acknowledged as significant obstacles for the provision of cancer screening in developing countries.^14^, ^15^

Financing counts a great deal for the sustainability of screening. Different countries have applied specific screening financing measures. Japan stipulates that the cost of gastric cancer screening is subsidized by the government, with a 30% co‐payment rate personally.^16^ South Korea's government and National Health Insurance (NHI) has been free of charge for Medicaid participants and those within the lower 50% of the NHI Corporation premium, although those within the upper 50% need to pay 10% co‐payment.^17^ England's National Health Service provides free breast, cervical, and colorectal cancer screening for the common mass.^18^ China's central transfer payment fund that is only free to a few areas has been the main screening financing resource, whereas most people need to pay their expenses during screening. But Screening costs that are fully biased to one side would prove unaffordable form for whether individuals or the government. Currently, most developing countries have not yet formed reasonable financing strategies. It is unclear for the cost‐sharing among financing resources including government, insurance, and individuals. Our study that ascertained the most appropriate proportion of individual payment takes the first step.

Willingness to pay (WTP) has been used extensively to obtain the amount of money that individuals will agree to pay for a product or service in many diverse settings.^19^ Specific methods to measure WTP include Contingent Valuation Method (CVM), Choice Experiments (CE), Conjoint Analysis (CA), etc. CVM, the most popular method to measure WTP, usually adopts a questionnaire survey to guide people's maximum WTP for goods or services by a hypothetical scenario, which can overcome the limitation of lacking market trading of public goods or new services to make researchers understand the public preference and then evaluate the value within various assumptions.^20^ Therefore, CVM was selected as the methodological tool to guide the WTP in this study.

Recently, a few studies have assessed populations' acceptance and WTP toward single‐site cancer screening such as colorectal, breast, upper gastrointestinal cancer, et al, which stated the high acceptance and WTP and relatively low WTP values for cancer screening. And factors including age, education level, income and cancer‐screening experience were related to residents' willingness to accept and pay for screening.^21^, ^22^, ^23^ However, there are limited studies focusing on individuals' acceptance and WTP toward multiple‐cancer packaging screening.

This paper aimed to examine rural residents' acceptance and WTP for packaging screening of 6 high risk cancers (that is lung, breast, colorectal, liver, gastric, and esophageal cancer), which will help to improve the coverage and sustainability of screening, and provide a reference basis for other developing countries to formulate reasonable screening strategies.

## 2 METHODS

### 2.1 Study population

This study took the rural areas of Shandong Province in eastern China as the research site. Using stratified random sampling, considering the economic development level and geographical location, Linqu, Feicheng, and Dongchangfu were selected as survey points in the East, Middle, and West of Shandong, respectively, and then rural residents in 2–5 villages were randomly selected from each county for the face‐to‐face questionnaire survey.

The respondents' inclusion criteria were as follows: (1) being aged 40–69 years old, because people aged 40 and above were high‐risk people of cancer, and people aged 70 and above were classified as the elderly whose understanding ability may affect the questionnaire quality; (2) had never been previously diagnosed with cancer.

The sample size of the study was calculated according to the following formula with the assumption of 50% willingness to pay (P), 95% confidence level, 5% margin of error.

$$N=\frac{Z_{\alpha /2}^{2}P\left(1-P\right)}{\varepsilon^{2}},$$

where Zα2=1.96, *P =* 50%, ε = 0.05.

Considering the 10% sample deletion rate, the sample size should be at least 423. In practice, we collected a larger sample size than expected to increase the reliability of the results.

### 2.2 Questionnaire design

The guidance tools of CVM include open‐ended, bidding game, payment card, single‐bounded dichotomous choices, and double‐bounded dichotomous choices. A combination of double‐bounded dichotomous choices and open‐ended questions was used to obtain accurate and realistic WTP values in the study.^19^ Scenario assumptions, as the core of CVM, were as follows in this study:

At present, the effective cancer‐screening methods include low‐dose spiral CT examination for lung cancer, endoscopy for gastric and esophageal cancer, serum alpha fetoprotein test combined with abdominal ultrasonography for liver cancer, fecal occult‐blood test (FOBT) and colono‐scopy for colorectal cancer, mammography, or ultrasonography‐screening procedure for breast cancer. Assuming that the local hospital can provide residents with the service of “packaging” six kinds of cancer screening simultaneously with the price of about ¥3000, what is your willingness to accept and pay?

**FIGURE 1:**
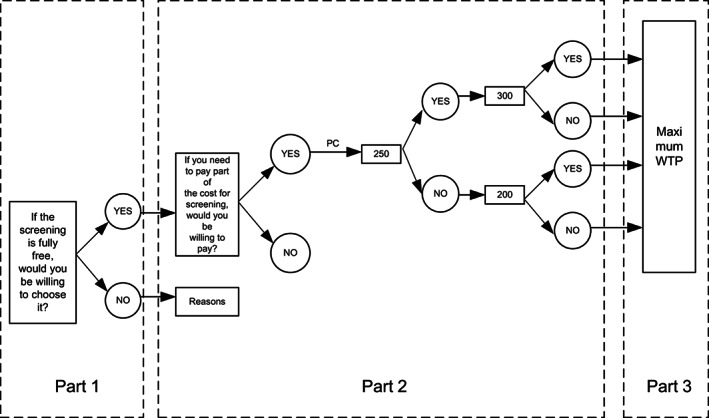
The process of inquiring respondents about their willingness to pay (WTP).

After introducing the scenario assumptions to the respondents, the first step was to ask the respondents whether they were willing to accept it in the case of the free PCS. The second step was to guide the WTP of the respondents who were willing to accept PCS by the method of double‐bounded dichotomous choices. According to our previous survey on the price of 6 high‐risk cancer screening, ¥3000 was finally determined as the total PCS price under the hypothetical scenario. The respondents were required to randomly select one from the price card (¥50, 150, 250, 350, 450, 600, 800, 950, 1200, 1800, 2200, and 2800, see Figure S1 for details) and then to be asked whether they were willing to pay the price on the card (intermediate value). If the answer was “YES”, the investigators inquired for a higher price, conversely, for a lower price (values on both sides). After two rounds, the double‐bounded dichotomous choices were completed. In the third step, an open‐ended question was used to obtain the respondents' MAXWTP (Figure 1).

### 2.3 Statistical analysis

The percentage of categorical variables was used to describe demographic characteristics, the chi‐square test was used to examine the between‐group difference with respect to demographic characteristics and willingness to accept PCS. Taking the acceptance for PCS (Yes or No) as the dependent variable and the statistically significant variables at the level of 0.05 in the Chi‐square test as the independent variables, multivariate logistic regression was used to explore the explanatory factors of respondents' willingness to accept PCS.

The determinants of WTP were analyzed with Tobit model (initial model), and then its marginal effect was obtained by incorporating the significant variables obtained from initial model into Tobit model again to eliminate the interference of confounding factors. Tobit model, as a censored regression model, is applicable to analyze a considerable number of data with zero dependent variables, which can explain the phenomenon of limited variable value and selection behavior. About two‐fifths of the participants in this study have WTP values of 0. Therefore, the Tobit model is the optimal model to analyze the determinants of WTP values, as shown below.

$${\mathrm{WTP}}_{i}^{*}=x_{i}^{\prime }\beta +\mu_{i},\mu_{i}\sim N\left(0,\sigma^{2}\right),$$

$${\mathrm{WTP}}_{i}=\left\{\begin{array}{c} {\mathrm{WTP}}_{i}^{*}\;\text{if}\;{\mathrm{WTP}}_{i}^{*}>0\;\\ 0\;\text{if}{\;\mathrm{WTP}}_{i}^{*}=0 \end{array}\right.,$$

where ${\mathrm{WTP}}_{i}^{*}$ is the unobserved latent variable, $x_{i}^{\prime }$ represents the independent variables, *β* is the regression coefficient of the independent variables, $\mu_{i}$ is the normally distributed error term.

Statistical tests were two‐sided using a 5% level of significance. All data were analyzed by Stata version 16.0.

## 3 RESULTS

### 3.1 Demographic characteristics

A total of 986 participants were surveyed, 959 were included after excluding the samples that did not meet the age criterion, including 320 residents in Feicheng City, 306 in Dongchangfu District, and 333 in Linqu County, with an effective rate of 97.26%. Table 1 shows that most participants were female (65.90%), over 50 years old (85.40%), married (95.72%), farmer (71.22%), with junior high school and below (87.38%), and had an annual income of 10,000–30,000 (57.04%). Besides, 95.93% of the residents had medical insurance for urban and rural residents, 35.45% had chronic diseases, 19.19% had cancer in their relatives, 31.49% had previous upper gastrointestinal symptoms, and 55.37% had cancer‐screening experiences.

**TABLE 1:** Demographic characteristics

Variable	Frequency	Percent (%)
Region		
Feicheng City	320	33.37
Dongchangfu District	306	31.91
Linqu County	333	34.72
Gender		
Male	327	34.10
Female	632	65.90
Age		
40–49	140	14.60
50–59	433	45.15
60–69	386	40.25
Education		
No schooling	156	16.27
Primary school	297	30.97
Junior high school	385	40.15
High school and above	121	12.62
Marriage		
Married	918	95.72
Single/divorced/widowed	41	4.28
Occupation		
Government, private enterprises, or workers	48	5.01
Self‐employed or freelance work	93	9.70
Farmer	683	71.22
Unemployed	104	10.84
Others	31	3.23
Annual income		
<10,000	208	21.69
10,000–30,000	547	57.04
>30,000	204	21.27
Medical insurance type		
Urban employee medical insurance	24	2.50
Medical insurance for urban and rural residents	920	95.93
Others^a^	15	1.56
Physical condition		
Health	619	64.55
Have chronic diseases	340	35.45
Cancer in relatives		
Yes	184	19.19
No	775	80.81
Previous upper gastrointestinal symptoms		
Yes	302	31.49
No	657	68.51
Cancer screening		
Yes	531	55.37
No	428	44.63
Total	959	100.00

^a^ Commercial medical insurance and no medical insurance.

### 3.2 WTP for packaging cancer screening

Table 2 shows 89.36% of participants were willing to accept PCS, comprising 57.66% of positive WTP and 31.70% of 0‐WTP. And 10.64% were unwilling to accept it, which the dominant reason being “attending clinics after feeling uncomfortable” (74.51%), followed by “other reasons” and “worrying about the pain or danger of examination” (17.65% versus 15.69%, respectively).

**TABLE 2:** WTP for packaging cancer screening

Variable	Frequency	Percent (%)
WTP values		
Protest‐0^a^	102	10.64
True‐0^b^	304	31.70
WTP >0	553	57.66
Reason for “Prorest‐0”		
Worry about the pain or danger of examination	16	15.69
Attend clinics after feeling uncomfortable	76	74.51
Physical examination organized by the work unit	2	1.96
Others^c^	18	17.65

Abbreviation: WTP, willingness to pay.

^a^ Unwilling to accept PCS.

^b^ Willing to accept PCS at zero money.

^c^ Others include unbelieving, unwillingness due to being far away and trouble, being sick, and only wanting to do an endoscopy.

**FIGURE 2:**
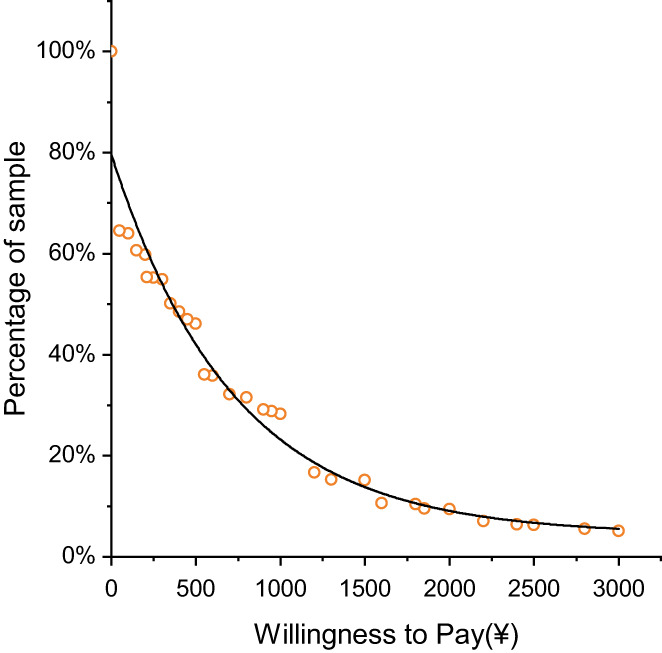
The demand curve for packaging cancer screening. The abscissa represents the WTP values for packaging cancer screening, ranging from ¥0–3000. WTP means the willingness to pay. The ordinate represents the proportion of residents' willingness to pay the corresponding price.

Figure 2 lists the distribution of participants' WTP values after excluding protest zeros. The values ranged from ¥50 to ¥3000. The respondents' average WTP for screening was ¥622, accounting for 20.73% of the total cost (¥3000). The WTP values went down as the bid prices increased. More than half of the residents (50.64%) were willing to pay less than ¥350, about one fifth (17.15%) were willing to pay less than ¥1200, and only 5.13% were willing to pay all ¥3000.

### 3.3 Associated factors for willingness to accept PCS

Table 3 shows that different regions, age, annual income, and cancer‐screening experiences had significant statistical differences in the acceptance of rural residents for PCS. Table 4 shows that region, age, annual income, and cancer‐screening experiences were significant factors. Participants living in Dongchangfu and Linqu were 74.9% and 85% less likely to accept PCS than those living in Feicheng, respectively (OR = 0.251, 95%CI: 0.113~0.557; OR = 0.150, 95%CI: 0.069~0.325). Participants aged 60–69 were 67.9% less likely to accept PCS compared with those aged 40–49 (OR = 0.321, 95%CI: 0.126~0.816). Participants with an annual income of ¥10,000–30,000 were 63.2% more inclined to accept PCS than those of less than ¥10,000 (OR = 1.632, 95%CI: 1.003~2.656). Besides, residents who had done cancer screening were 58.1% more inclined to accept PCS than those who had not (OR = 0.581, 95%CI: 0.371~0.909).

**TABLE 3:** Univariate analysis for willingness to accept packaging cancer screening

Variable	Acceptance		*χ* ^2^	*p*
	Yes (*n* = 857)	No (*n* = 102)		
Region			36.69	<0.001
Feicheng City	312 (97.50)	8 (2.50)		
Dongchangfu District	268 (87.58)	38 (12.42)		
Linqu County	277 (83.18)	56 (16.82)		
Gender			0.002	0.961
Male	292 (89.30)	35 (10.70)		
Female	565 (89.40)	67 (10.60)		
Age			10.91	0.004
40–49	134 (95.71)	6 (4.29)		
50–59	391 (90.30)	42 (9.70)		
60–69	332 (86.01)	54 (13.99)		
Education			4.61	0.203
No schooling	132 (84.62)	24 (15.38)		
Primary school	268 (90.24)	29 (9.76)		
Junior high school	349 (90.65)	36 (9.35)		
High school and above	108 (89.26)	13 (10.74)		
Marriage			0.72	0.396
Married	822 (89.54)	96 (10.46)		
Single/divorced/widowed	35 (85.37)	6 (14.63)		
Occupation			6.11	0.191
Government, private enterprises, or workers	43 (89.58)	5 (10.42)		
Self‐employed or freelance work	84 (90.32)	9 (9.68)		
Farmer	616 (90.19)	67 (9.81)		
Unemployed	90 (86.54)	14 (13.46)		
Others	24 (77.42)	7 (22.58)		
Income			9.54	0.009
<10,000	174 (83.65)	34 (16.35)		
10,000~30,000	495 (90.49)	52 (9.51)		
>30,000	188 (92.16)	16 (7.84)		
Medical insurance type			1.07	0.585
Urban employee medical insurance	20 (83.33)	4 (16.67)		
Medical insurance for urban and rural residents	824 (89.57)	96 (10.43)		
Others^a^	13 (86.67)	2 (13.33)		
Physical condition			0.03	0.8545
Health	554 (89.50)	65 (10.50)		
Have chronic diseases	303 (89.12)	37 (10.88)		
Cancer in relatives				
Yes	166 (90.22)	18 (9.78)	0.17	0.676
No	691 (89.16)	84 (10.84)		
Previous upper gastrointestinal symptoms			0.86	0.353
Yes	274 (90.73)	28 (9.27)		
No	583 (88.74)	74 (11.26)		
Cancer Screening				
Yes	493 (92.84)	38 (7.16)	15.16	<0.001
No	364 (85.05)	64 (14.95)		

^a^ Commercial medical insurance and no medical insurance.

**TABLE 4:** Multivariate logistic regression for willingness to accept packaging cancer screening

Variable	Coefficient	SE	*t*‐value	*p*‐value	OR	95%CI
Region (ref. Feicheng city)						
Dongchangfu District	−1.383	0.416	−3.40	0.001**	0.251	0.113, 0.557
Linqu county	−1.896	0.396	−4.82	0.000**	0.150	0.069, 0.325
Age (ref. 40–49)						
50–59	−0.709	0.458	−1.53	0.125	0.492	0.199, 1.218
60–69	−1.136	0.465	−2.39	0.017*	0.321	0.126, 0.816
Annual income (ref. <10,000)						
10,000–30,000	0.49	0.25	1.97	0.049*	1.632	1.003, 2.656
>30,000	0.377	0.35	1.05	0.292	1.458	0.723, 2.94
Cancer screening (ref. Yes)						
No	−0.544	0.233	−2.38	0.017*	0.581	0.371, 0.909

Abbreviations: CI, confidence interval; SE, standard error.

* *p* < 0.05

** *p* < 0.01.

### 3.4 Associated factors of WTP for PCS and the margin effects

Referring to prior studies,^24^ protest‐0 responses were excluded from the Tobit model in the study. Table 5 shows that participants who lived in Linqu had lower WTP than those living in Feicheng (*t*‐value = −2.84). The margin effect result shows that the participants living in Linqu were willing to pay ¥175.163 less than those living in Feicheng. For those with positive WTP, participants living in Linqu were willing to pay ¥125.388 less than those living in Feicheng.

**TABLE 5:** Tobit regression for willingness to pay and the margin effects

Parameter for MWTP	Coefficient	SE	*t*‐value	*p*‐value	Marginal effect = probability (d*y*/d*x*)	
					WTP > 0	WTP ≥ 0
Region (ref. Feicheng city)						
Dongchangfu District	106.59	105.367	1.01	0.312		
Linqu county	−293.498	103.38	−2.84	0.005**	−125.388	−175.163
Gender (ref. Male)						
Female	−323.756	94.162	−3.44	0.001**	−156.381	−219.290
Age (40–49)						
50–59	−178.421	117.647	−1.52	0.13		
60–69	−248.881	133.715	−1.86	0.063		
Education (ref. No schooling)						
Elementary school	−149.761	134.24	−1.12	0.265		
Junior high school	19.245	139.594	0.14	0.89		
High school and above	39.719	174.393	0.23	0.82		
Marriage (ref. Married)						
Single/divorced/widowed	−183.92	211.874	−0.87	0.386		
Occupation (ref. Government, private enterprises or workers)						
Self‐employed or freelance work	457.5	219.411	2.09	0.037*	194.305	271.938
Farmer	212.842	192.15	1.11	0.268		
Unemployed	363.627	226.608	1.60	0.109		
Others	75.323	295.312	0.26	0.799		
Income (<10,000)						
10,001–30,000	358.413	108.911	3.29	0.001**	162.152	226.009
>30,000	425.646	137.159	3.10	0.002**	240.755	336.725
Medical insurance type (ref. urban employee medical insurance)						
Medical insurance for urban and rural residents	−345.529	261.831	−1.32	0.187		
Others^a^	−886.262	457.884	−1.94	0.053		
Physical condition (ref. Health)						
Have chronic diseases	−77.217	86.111	−0.90	0.37		
Cancer in relatives (ref. Yes)						
No	−71.589	100.665	−0.71	0.477		
Previous upper gastrointestinal symptoms (ref. Yes)						
No	−35.405	87.487	−0.40	0.686		
Cancer screening (ref. Yes)						
No	−246.655	87.866	−2.81	0.005**	−103.599	−144.862

Abbreviations: MWTP, maximum values of WTP; SE, standard error.

^a^ Commercial medical insurance and no medical insurance.

* *p* < 0.05

** *p* < 0.01.

Female was significantly correlated with lower WTP at a level of 0.05 (*t*‐value = −3.44). Female was willing to pay ¥219.290 less than male, and female with positive WTP was willing to pay ¥156.381 less than male.

The WTP of self‐employed or freelancers was higher than that of participants from government, enterprises, or factories (*t*‐value = 2.09). The self‐employed or freelancers were willing to pay ¥271.938 more than those from government, private enterprises, or factories.

Income had a significantly positive effect on WTP (*p* < 0.05), indicating that participants with higher income were more willing to pay more for cancer screening. Participants with an income of ¥10,001–30,000 and over ¥30,000 were more willing to pay ¥226.009 and ¥336.725 than those with an income of less than ¥10,000, respectively. For participants with WTP >0, those with income of ¥10,001–30,000 and over ¥30,000 were willing to pay ¥161.152 and ¥240.755 more than income of less than ¥10,000, respectively.

Likewise, cancer‐screening experiences had a significantly positive impact on WTP at a level of 0.05 (*t*‐value = −2.81), indicating that participants who had cancer screening were more willing to pay more. Participants who had cancer screening were willing to pay ¥144.862 more than those without.

## 4 DISCUSSION

This study, as far as we know, is the first to examine residents' acceptance and WTP for packaging cancer screening. Rural residents have a high acceptance (89.36%), which reflects the possibility of popularizing PCS to a certain extent. Despite the provision of free cancer screening, 10.64% of respondents indicated reluctance to accept it. The main reason was that they did not attend clinics until feeling uncomfortable, but it was too late by then, which stated the necessity to strengthen rural residents' awareness and enthusiasm for precancerous screening. Besides, respondents placed more worry on the pain or danger of examination, as with the results of colonoscopy‐related packaging screening.^25^ It appeared that standardized training, operation skill, and comfortable processes should be emphasized and strengthened.

Region, age, annual income, and cancer‐screening experiences were the significant influencing factors of residents' acceptance for PCS. Residents in Dongchangfu and Linqu, with higher age (60–69), with lower income (<10,000) and without cancer‐screening experiences had lower acceptance of PCS, which may be related to their weak health awareness, poor cognition and understanding of health information, slender family income, and limited previous cancer‐screening experience. It suggested that these kinds of population may be the key intervention population, and efforts should be made to promote cancer screening‐related health education to the public on the severity of cancer, the importance of screening, and provide screening costs subsidies to rural residents to a certain extent, so as to improve the population‐screening participation rate.^22^, ^26^

And 57.66% of respondents were willing to pay for PCS, lower than 76.7% in urban China,^14^ 88% in England,^27^ 72% for breast cancer screening in America,^21^ implying that rural residents' WTP seemed to be relatively low. A study on the attitude of rural residents toward cancer screening in China concluded that most rural residents were only willing to pay 10%~30% of the total screening cost, whether single‐site cancer screening or multiple‐site packaging cancer screening.^28^ Our study used CVM to derive the specific WTP values of rural residents, which may be closer to the actual situation. The results show that the average WTP of respondents was ¥622, accounting for 20.73% of the total cost of ¥3000, suggesting that about 20% may be the optimal payment proportion to maximize rural residents' participation in packaging cancer screening. And more than half of respondents were willing to pay only ¥350, which put far from meeting the actual charge of ¥2000–3000 in local hospitals. In other words, rural residents expressed a relatively small amount of WTP in addition to the low WTP. Combined with various studies on cancer screening, price is an important obstacle to WTP.^29^, ^30^ Whereas cancer‐screening costs are usually fully paid by the demander in the case that the current government and health insurance have not yet taken action on mass cancer screening.^31^ Because the USA has incorporated cancer screening into Medicare, the cancer‐screening rate of those with Medicare was reported significantly higher than those without.^32^ Again, a study in the USA pointed out that Medicaid reimbursement was significantly correlated with the screening participation rate.^33^ Studies in Japan found that reducing out‐of‐pocket costs can arouse breast and cervical cancer screening attendance.^34^, ^35^ Thus, we encourage the government and health insurance to participate in the payment of PCS and manage to bear the remaining 80% except about 20% of the screening fees that individuals were willing to pay themselves. In the future, multi‐party financing for cancer screening (e.g. individuals, insurance, and government) may become a promoted policy for rural residents to undergo PCS.

Male, cancer‐screening experiences and higher income affected WTP values positively. It may be related that residents with the above characteristics can afford cancer‐screening costs based on meeting basic needs, coupled with their high acceptance of screening forms and technology. These findings were roughly consistent with the study in China,^23^, ^31^ England on colorectal cancer,^36^ and Ethiopia on cervical cancer.^29^ Besides, the region was one of the factors affecting WTP, of which Feicheng residents were willing to pay more for PCS than Linqu residents. It may be because Feicheng has just organized endoscopic screening before our survey, which further confirmed the importance of cancer‐screening experience to residents' WTP. Moreover, it was interesting that self‐employed or freelancers would pay more than the participants from government, enterprises, or factories, with the reverse being the case for Zhou's study.^23^ The possible explanation was that these two occupations accounted for a small proportion of our study, so this result should be interpreted with caution. The above findings suggest that it is necessary to strengthen publicity and raise public awareness of cancer screening. More importantly, the economic level greatly limits WTP, which also certifies the importance to broaden financing channels to improve cancer‐screening utilization.

The present study has some limitations. First, the respondents were from Shandong and interviewed during a specific period, thus the results need to be carefully applied to the whole rural residents in China. Second, given the hypothetical nature, the WTP values answered by respondents may differ from the corresponding actual behavior. To improve the quality and effectiveness of the findings, the connection between willingness and actual behaviors should be examined in future studies. Third, this study only discussed the influencing factors of WTP on the side of demographic characteristics. Future research should involve participants' knowledge of cancer screening and their health behavior.

## 5 CONCLUSION

Rural residents were willing to accept and pay for PCS, but the WTP values were low. Region, age, annual income, and cancer‐screening experiences were the significant influencing factors of residents' acceptance for PCS. And Feicheng residents, male gender, self‐employed or freelancers, residents with higher income and cancer‐screening experience had higher willingness to pay for PCS. On the one hand, to improve public awareness and participation in precancerous screening, it is necessary to carry out packaging cancer‐screening publicity activities. On the other hand, for improving the coverage and sustainability of cancer screening, residents' out‐of‐pocket proportion should be controlled within 20% and the rest could be borne by the government, insurance, and other sources. Additionally, should the next research discuss the providers' WTP for PCS in combination with our findings, it will be more conducive to format a reasonable financing mechanism.

## AUTHOR CONTRIBUTIONS

Zhang Q: Data curation, Formal analysis, Methodology, Software, Writing‐original draft; Ren D: Data curation, Investigation, Software; Chang X: Data curation, Investigation; Sun C: Data curation, Investigation; Liu R: Data curation, Investigation; Wang J: Funding acquisition, Project administration, Resources, Supervision, Writing‐review & editing; Zhang N: Conceptualization, Funding acquisition, Investigation, Project administration, Resources, Supervision, Writing‐review & editing.

## FUNDING INFORMATION

This study was supported by the National Natural Science Foundation of China (71904109) and the Natural Science Foundation of Shandong Province (ZR2019PG006).

## CONFLICT OF INTEREST

None.

## ETHICS APPROVAL STATEMENT

This study was ethically approved by the Institutional Ethical Review Board of Shandong Cancer Hospital and Institute (SDTHEC201909001).

## INFORMED CONSENT STATEMENT

All participants gave written informed consent prior to engaging in this study.

## Supporting information


Figure S1

## Data Availability

The data that support the findings of this study are available from the corresponding author upon reasonable request.
