# Supplementary material for: Willingness to pay for packaging cancer screening of Chinese rural residents
Source: Cancer Med. 2022 Aug 24;12(3):3532–42. doi: 10.1002/cam4.5162 (PMC9939105; doi:10.1002/cam4.5162)
Supplement: Supplementary file 1 — Figure S1 [file CAM4-12-3532-s001.docx]

**Supplementary Materials**

1 50 100

200 250 300

100 150 200

500 600 700

400 450 500

300 350 400

1000 12001500

900 950 1000

700 800 900

2500 28003000

2000 22002500

1500 18002000

**S1 Fig.** Price Card
